# A Novel Method for Electrophysiological Analysis of EMG Signals Using MesaClip

**DOI:** 10.3389/fphys.2020.00484

**Published:** 2020-06-09

**Authors:** Lukasz Wiklendt, Simon J. H. Brookes, Marcello Costa, Lee Travis, Nick J. Spencer, Phil G. Dinning

**Affiliations:** ^1^College of Medicine and Public Health, Flinders University, Bedford Park, SA, Australia; ^2^Department of Surgery, Flinders Medical Centre, Bedford Park, SA, Australia

**Keywords:** artifact removal, non-harmonic model, action potential, wavelet transform, time-frequency analysis

## Abstract

In electrophysiology, many methods have been proposed for the analysis of action potential firing frequencies. The aim of this study was to present an algorithm developed for a continuous wavelet transform that enables the filtering out of frequencies contributing to the shapes of action potentials (spikes), whilst retaining the frequencies that encode the periodicity of spike trains. The continuous wavelet transform allows us to decompose a signal into its constituent frequencies. A signal with a single event, such as a spike, is composed of frequencies that characterize the shape of the spike. A signal with two spikes will also be composed of frequencies characterizing the shape of the action potential, but in addition will include a substantial portion of its power at the frequency corresponding to the time-difference between the two spikes. This is achieved by clipping peaks from the wavelet amplitudes that are narrower than a given minimum number of phase cycles. We present some application examples in both synthetic signals and electrophysiological recordings. This new approach can provide a major new analytical tool for analysis of electrophysiological signals.

## 1. Introduction

In electrophysiology, non-stationary quasiperiodic time series signals are common. The continuous wavelet transform (Mallat, 2008) is a useful tool for analysing such signals. It decomposes a time domain signal *x*(*t*) into the time-scale domain *w*(*t, s*), by cross-correlating *x*(*t*) with a wavelet function ψ_*s*_(*t*) that contains an intrinsic frequency *f*_*s*_ inversely proportional to scale *s* and localized about time *t* = 0. Intuitively, *w*(*t, s*) is a measure of the strength of oscillation of *x*(*t*) at a frequency *f*_*s*_ within a neighborhood at time *t* of duration proportional to *s*.

The wavelet transform is ideally suited to measuring the time-varying frequencies of smoothly oscillating signals. However, in many cases, signals could be described as more “spiky” than “smooth.” A spiky signal is characterized by short-duration changes in amplitude separated by much longer quiescent periods, where each short-duration change is considered an event of interest and referred to as a “spike.” For such signals, even though the wavelet transform does capture the frequency at which the spikes occur, it also produces harmonic artifacts that capture the higher frequency changes characterizing the shape of each individual spike.

The conventional approach for measuring the frequency of spiky events is to identify the events via peak-detection (e.g., Nenadic and Burdick, 2005) and report the intervals between adjacent-in-time events. The main advantage of such an approach is that the precise locations-in-time of each event are found, and so the precise intervals between events can be obtained. In some application domains, such as spike sorting (Lewicki, 1998), event times as well as the event shapes need to be identified so that the signal can be decomposed into its additive component signals based on the shapes of events, and then the event frequencies calculated for each signal separately. Precise event times also allow for the inference of parameters of non-periodic activity, for example, the instantaneous rate of a Poisson point process (Adams et al., 2009). In such cases, the algorithm presented in this paper would be of limited use.

The algorithm presented here (in Algorithm 1), which we call “mesaclip,” does not identify individual events. Rather it clips amplitudes from the time-scale representation of a signal that span a period of time too short to contribute to multiple events. This is done by removing amplitudes from the result of a wavelet transform that are shorter than *k* cycles, where *k* is the algorithm's only parameter and *k* = 2 is an excellent default.

It should be noted that the purpose is not to remove spike data from the original time-domain signal, such as described in Ehrentreich and Sümmchen (2001) or Veneri et al. (2011). Retaining the spikes is essential to recovering the frequency of those events. An input spike event will have a transient “peaked” response at higher frequencies, and it is this response that is clipped, while the event's contribution to lower frequencies is retained, exposing the frequency of a train of such events.

A motivation for using the wavelet transform in conjunction with mesaclip, over peak detection, is the ability to reveal multiple simultaneous frequencies in a signal, such as the frequency of recurring bouts of events. Also, the phase of the signal at each frequency is retained, allowing for the phase-difference to be calculated against a second signal via the *cross wavelet transform*, a technique useful for computing interactions between two or more signals (Torrence and Webster, 1999; Bloomfield et al., 2004; Grinsted et al., 2004).

A recent approach, called *de-shape*, for removing the influence of the wave-shape from the time-frequency response is presented in Lin et al. (2018). It appears to have similar qualitative results to the algorithm presented in this paper. There, the short-time Fourier transform (STFT) is multiplied by the inverse short-time cepstral transform (iSTCT). The STFT contains the harmonic series, that is, integer multiples of the fundamental frequency, and the iSTCT contains a *sub-harmonic* series, which are integer divisions of the fundamental frequency. Multiplying the two removes the harmonics and retains the fundamental frequency.

This paper is organized as follows: section 2 gives a brief overview of the wavelet transform, section 3 describes the mesaclip algorithm that is the main contribution of this paper, section 4 presents results on real and synthetic signals, section 5 is a discussion of the results, limitations, and advantages, and section 6 concludes the paper.

## 2. Wavelet Transform

The wavelet transform converts a signal from the time-domain to the time-frequency domain, such that for each point in time there is a decomposition of the signal around that point in time into its constituent frequencies. More precisely, the transform converts to a time-scale domain, but appropriate mapping from scale to frequency can be chosen.

For computing the wavelet transform on an input signal *x*(*t*) ∈ ℝ, we choose a wavelet basis function ψ(*t*) ∈ ℂ and set of positive time scales *S* = {*s*_1_, …*s*_*L*_}. An admissible wavelet function is one which has zero mean and its Fourier transform is continuously differentiable (Farge, 1992), with an extra desirable property that it be localized in both time and frequency. For each scale, a wavelet is constructed by effectively stretching in time the wavelet basis function by that scale, while normalizing to unit energy (1).

(1) $$\begin{array}{r} \psi_{s}\left(t\right)=\frac{1}{\sqrt{s}}\psi \left(\frac{t}{s}\right) \end{array}$$

(2) $$\begin{array}{r} w_{s}\left(t\right)=\left(x⋆\psi_{s}\right)\left(t\right) \end{array}$$

To compute the transform, for each scale *s* ∈ *S*, we compute the cross-correlation of the signal with the wavelet (2). A finite set of scales is explicitly chosen here, since the algorithm presented in this paper operates on each scale independently, and to formalize this we've moved the scale argument into a subscript *w*(*t, s*) → *w*_*s*_(*t*). The resulting output is a time-scale representation of the signal, which can be mapped to a time-frequency representation by the reciprocal function *f* = *c*/*s*, for frequency *f*, scale *s*, and a ψ-dependent constant factor *c*. See Torrence and Compo (1998) or Mallat (2008) for an introduction to wavelet analysis.

Another way to map from scales to frequencies is via synchrosqueezing (Daubechies et al., 2011). Synchrosqueezing calculates the frequency not as a function of the scale but by redistributing the wavelet amplitudes based on the time-derivative of the phase (i.e., instantaneous frequency). This approach is included in the examples presented in this paper, although it is applied as a post-process to the mesaclip algorithm, that is, the redistribution of amplitudes occurs after they have been clipped.

## 3. MesaClip

The result of the wavelet transform, for a scale *s* ∈ *S*, can be written in polar-form as *w*(*t*) = *r*(*t*)*e*^*iϕ*(*t*)^ for some time-varying amplitude *r* ∈ ℝ^≥0^ and phase ϕ ∈ ℝ (omitting explicit scale identification for brevity). An unwrapped phase ϕ is assumed, and that ϕ is non-decreasing $\frac{d\phi \left(t\right)}{dt}\geq 0$. If the phase is not non-decreasing, then we set negative instantaneous frequencies to 0, thereby enforcing monotonicity.

A difference in phase from time *t*_1_ to time *t*_2_ of ϕ(*t*_2_)−ϕ(*t*_1_) = 2π*k* means there are *k* cycles over that time interval in the signal at the given scale *s*. A substantially high peak in *r* over this time-range suggests a substantially strong oscillation. If the width of such a peak is localized over a short phase-range, ϕ(*t*_2_)−ϕ(*t*_1_) <2π*k*, then the peak spans fewer than *k* cycles. If we clip the amplitude of all peaks that are narrower-in-phase than some given constant 2π*k*, down to an amplitude such that the tops of resulting plateaus are of width 2π*k*, then we will have effectively removed amplitudes contributing to oscillations shorter than *k* cycles.

To illustrate the idea of clipping formally, consider a function *g*(φ) = *r*(ϕ^−1^(φ)) that maps from phases to amplitudes^1^. We can wrap additional scaffolding around *g* with

(3) $$\begin{array}{l} g(\varphi )=\text{max}\{h\;|\;(\exists [a,b]\subset_{\emptyset }\mathbb{R})(\forall \varphi^{\prime }\in [a,b])\\ \;\left[\varphi \in [a,b]\wedge r(\phi^{-1}(\varphi^{\prime }))\geq h\right]\}, \end{array}$$

which, for a given phase φ, finds the largest *h* such that there exists at least one non-empty phase interval [*a, b*] containing φ and where the amplitude at all elements of [*a, b*] is no less than *h*. Although (3) appears redundant, it allows us to now append the extra condition *b*−*a*≥κ, which specifies a minimum width κ for each interval, resulting in the function

(4) $$\begin{array}{l} g_{\kappa }(\varphi )=\text{max}\{h|\;(\exists [a,b]\subset_{\emptyset }\mathbb{R})(\forall \varphi^{\prime }\in [a,b])\\ \;\left[\varphi \in [a,b]\wedge r(\phi^{-1}(\varphi^{\prime }))\geq h\wedge b-a\geq \kappa \right]\}. \end{array}$$

For κ = 2π*k*, the function *g*_2π*k*_(φ) corresponds to one where the tops of narrow high peaks have been clipped away leaving lower plateaus of width no less than *k* cycles. Note, it is the peaks in the wavelet amplitudes that are clipped, not the spikes in the original time-domain signal.

**Figure 1:**
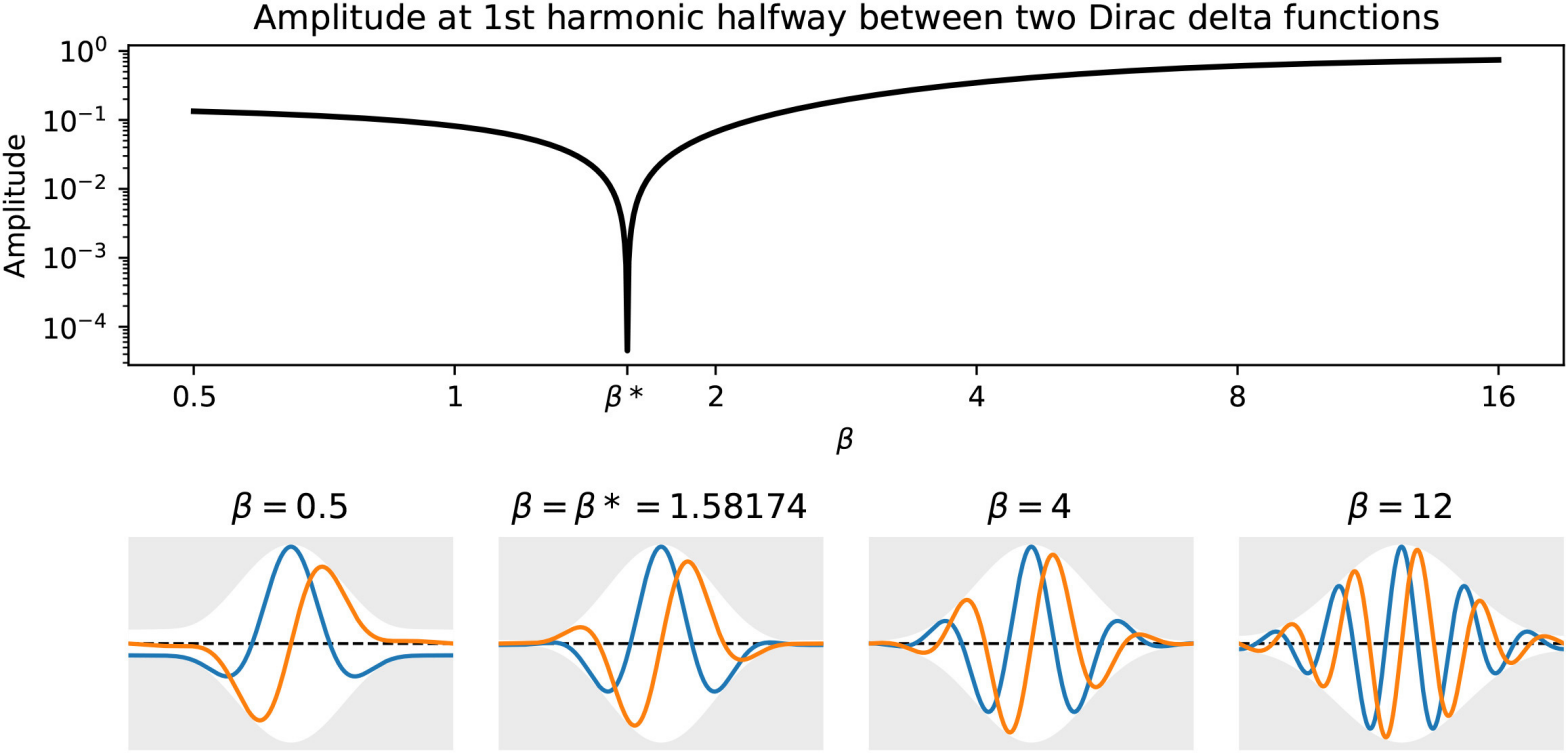
Given γ = 3, the top subplot shows the amplitude in (5) for a range of βs, with a clear minimum at the optimal β^*^ = 1.58174. The four bottom plots show time-domain versions of the Morse wavelet for a selection of βs, where gray outlines the amplitude envelope, and blue and orange curves represent the real and imaginary components of the wavelet.

A critical component of the algorithm is an appropriate choice of wavelet function. We use the Morse wavelet, which generalizes many conventional wavelet functions (Lilly and Olhede, 2012), and remains analytic even for highly time-localized instances. It admits two parameters, β and γ. We use a value of γ = 3 as it results in wavelets that are symmetric in frequency space and has a small Heisenberg area (Lilly and Olhede, 2009). We choose the value β = β^*^ heuristically, where the value β^*^ = 1.58174 minimizes (practically zeroes) the amplitude of the first harmonic of the wavelet transform of a Dirac comb, halfway between two successive Dirac delta functions. Since the discrete-time Fourier transform of a Dirac delta function is 1, then β^*^ can be calculated (5) by numerically minimizing, at the middle time sample *n* = *N*/2, the absolute value of the inverse discrete Fourier transform of the frequency-domain Morse wavelet basis function Ψ_β, γ_ scaled to a peak frequency of 2Hz,

(5) $$\begin{array}{r} \beta^{*}=\underset{\beta }{\mathrm{argmin}}|\text{FF}{\text{T}}^{-1}\left(\sqrt{s}\;{\text{Ψ}}_{\beta ,\gamma }\left(s\omega \right)\right)\left[n\right]| \end{array}$$

(6) $$\begin{array}{r} {\text{Ψ}}_{\beta ,\gamma }\left(\omega \right)=2{\left(e\gamma /\beta \right)}^{\beta /\gamma }\omega^{\beta }e^{-\omega^{\gamma }} \end{array}$$

(7) $$\begin{array}{r} s=\frac{\omega_{\beta ,\gamma }}{2\pi }\frac{1}{2} \end{array}$$

(8) $$\begin{array}{r} \omega_{\beta ,\gamma }={\left(\beta /\gamma \right)}^{1/\gamma } \end{array}$$

where ω represents an array of *N* frequencies from 0 to 2π(*N*−1) radians. Figure 1 shows the amplitude in (5) for β ranging from 0.5 to 16, with a clear minimum at β = 1.58174.

Now that a general description of the desired effect has been outlined, the following introduces the algorithm in detail. Mesaclip takes two real-valued arrays *r* and ϕ, and a real-valued scalar parameter κ. In the context of our problem domain, the parameter will be set to κ = 2π*k*, where *k* represents a number of cycles. The array *r* contains the wavelet amplitudes and ϕ contains their unwrapped non-decreasing phases, for any given scale. The algorithm works by iterating over peaks in *r*, and for each peak expanding a range of indexes represented by the pair of inclusive end-indexes (*a, b*), reinitialized each time we advance to a new peak index. Either the range expands to a sufficient width, ϕ[*b*]−ϕ[*a*]≥κ, such that it can be clipped with *r*[*a*…*b*]←min(*r*[*a*], *r*[*b*]), or a trough is reached at one of the range's ends in which case either the range can be combined with a previous range or it is pushed onto a stack of previous ranges for later combination. Once all peaks have been processed in this way, the peaks located at any remaining ranges on the stack are clipped. See Algorithm 1 for extra detail including edge cases. The time-complexity of mesaclip is *O*(*n*), and it has practically negligible impact on the performance of the overall wavelet analysis.


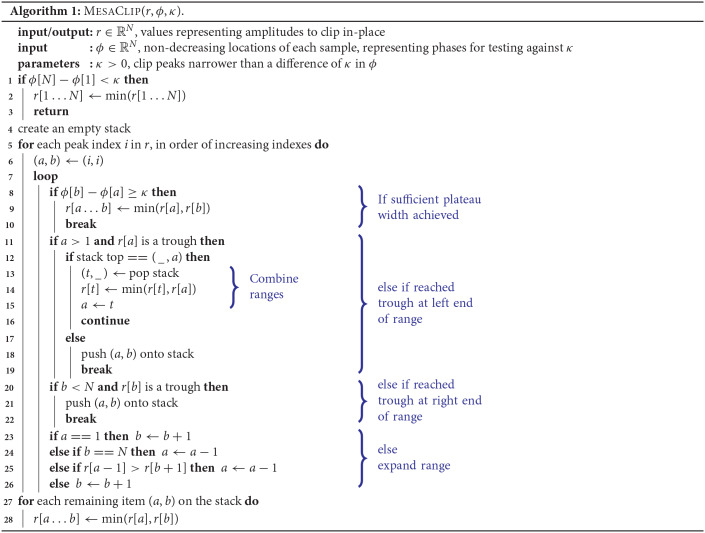


**Figure 2:**
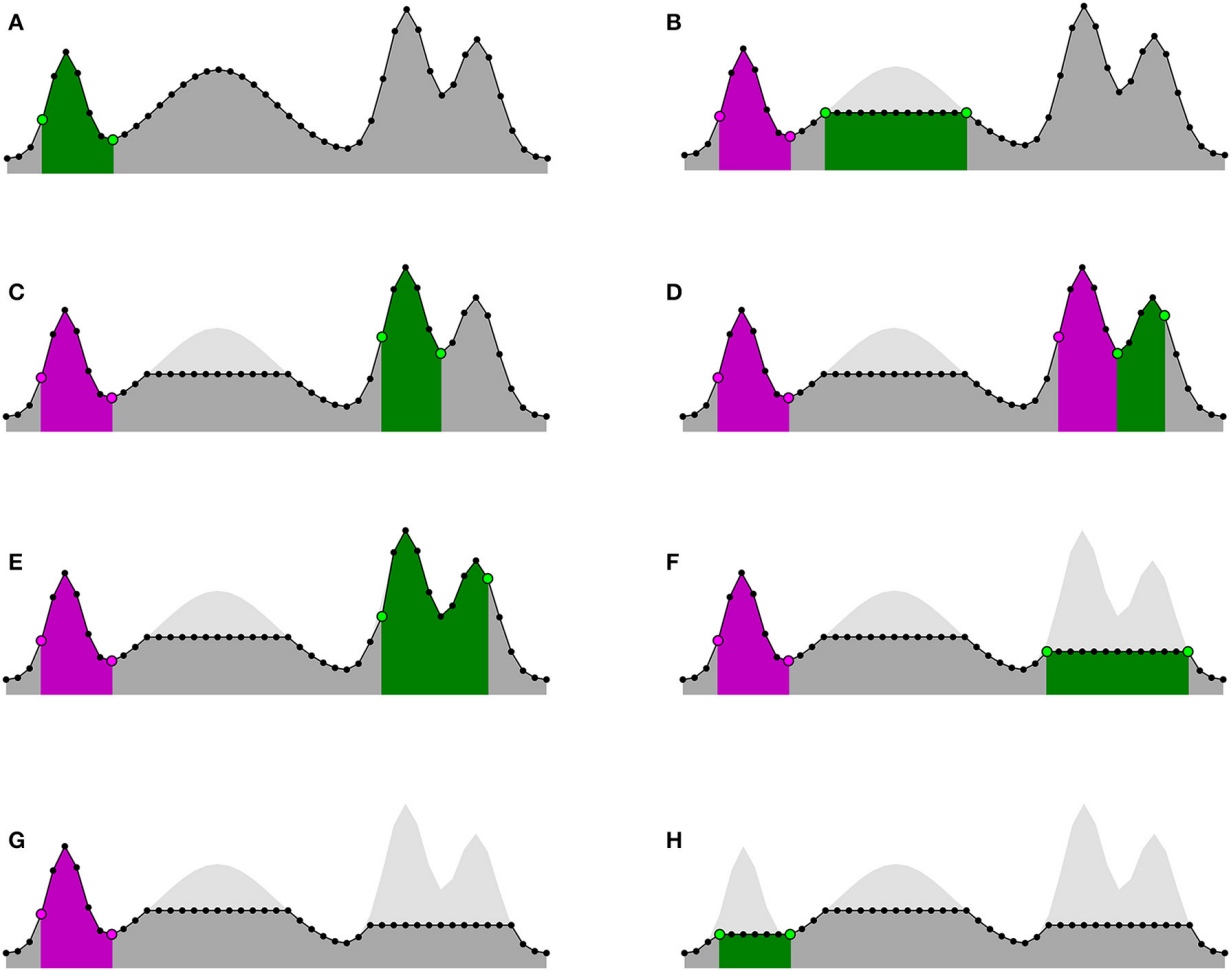
Execution of mesaclip on an example input with 4 peaks, with parameter κ = 12, and for ϕ a uniformly increasing array of indexes giving horizontal locations left-to-right, and *r* plotted as the height. Dots represent the 47 individual array elements in ϕ and *r*. Sub-captions reference the algorithm line-numbers in bold. Light-gray represents the original input *r*, and dark-gray represents the current state of the clipped *r*. Green represents the current range “(*a, b*),” and magenta represents ranges on the stack. **(A)** First peak reached a trough on the right-end of its range, and is placed on the stack (**20**–**21**). **(B)** Second peak achieved the sufficient width, and is clipped (**8**–**9**). **(C)** Third peak reached a trough on the right-end of its range, and is placed on the stack (**20**–**21**). **(D)** Fourth peak reached a trough on the left-end of its range (**11**), and a touching range is found on top of the stack (**12**) …. **(E)** …so the two ranges are combined (**13**–**15**) into a single range, which then continues to expand in the (**7**) loop. **(F)** The combined range of the third and fourth peaks finally reaches the sufficient width, and is clipped (**8**–**9**). **(G)** All peaks have now been processed, terminating the (**5**) for-loop and moving on to (**27**). **(H)** Each range remaining on the stack is clipped (**27**–**28**).

Figure 2 depicts some key moments in the execution of mesaclip, where the values of ϕ are uniformly increasing for the purpose of simplifying the illustration, but in practice they would correspond to a non-uniformly increasing phase.

## 4. Results

**Figure 3:**
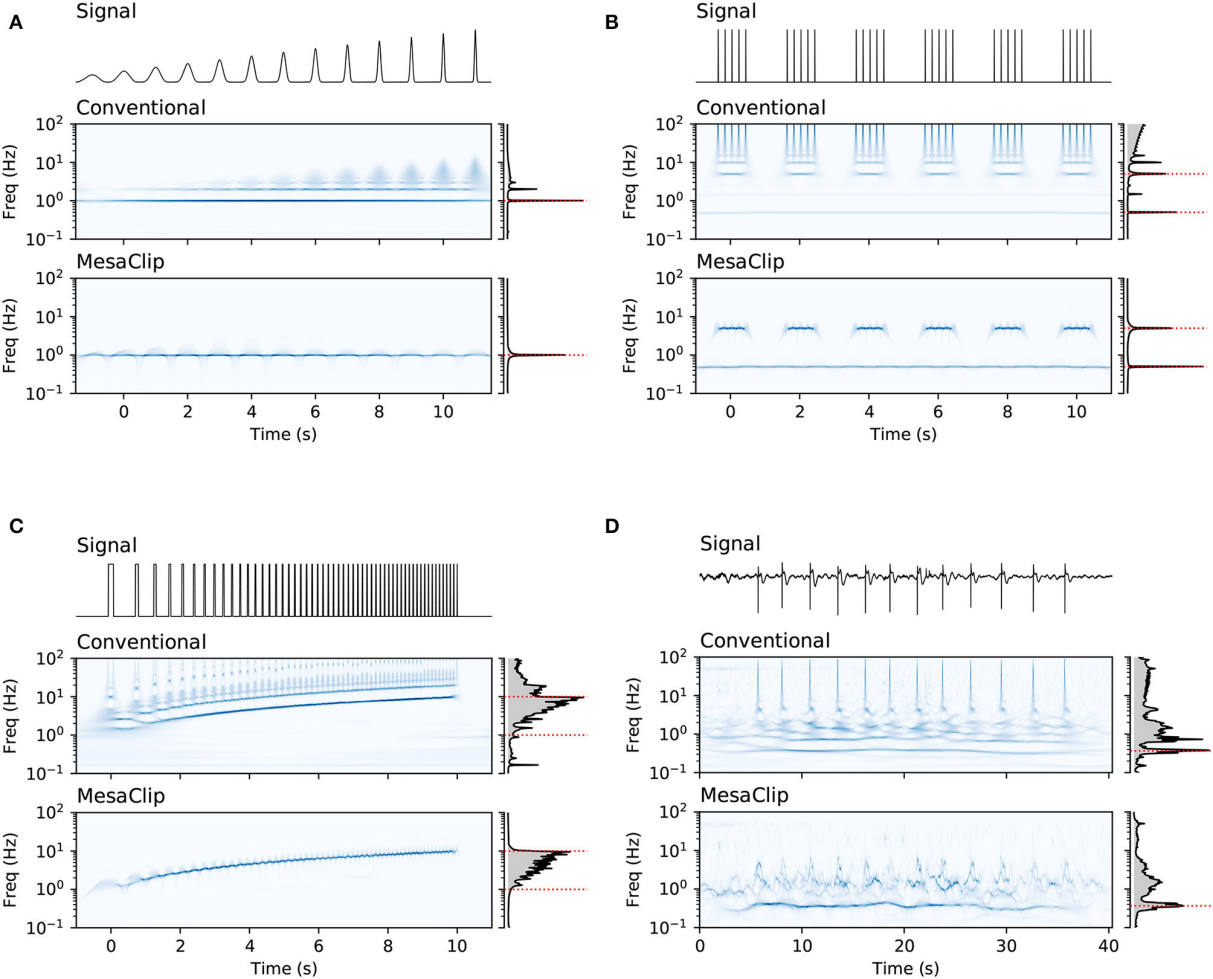
Signals are shown in black above each of the four subplots. The middle boxes depict the wavelet amplitudes for a conventional Morse (β = 12, γ = 3) wavelet transform approach. The bottom boxes show the amplitudes for the mesaclip approach with *k* = 2 and a Morse (β = 1.58174, γ = 3) wavelet. Synchrosqueezing is applied in both approaches as a post-process. The global wavelet spectrum is drawn to the right of each box, with horizontal red dotted lines annotating true frequency properties of the signal. **(A)** A constant 1Hz signal gradually morphing from smooth oscillation to a spiky signal. Harmonic artifacts at 2, 3 Hz, and higher creep into the conventional wavelet approach, but are missing in the mesaclip approach. **(B)** A bursting spiky signal, showing mesaclip removes harmonic artifacts while retaining both spiking and bursting frequencies. **(C)** A thresholded chirp gradually increasing from 1 to 10 Hz, showing mesaclip can handle a varying frequency. **(D)** A signal obtained from a real EMG recording of smooth muscle. The positive and negated negative components are transformed separately and their results are summed. The horizontal red dotted line corresponds to the frequency obtained by inverting the average inter-spike-interval of the 12 clear spikes. The conventional approach suffers from harmonic artifacts not present in the mesaclip approach.

To illustrate the effect of the mesaclip approach compared to a conventional wavelet transform approach, Figure 3 shows three artificial signals (a-c) and one real EMG signal recorded from smooth-muscle (d). A Python implementation is available at https://github.com/lwiklendt/mesaclip.

**Figure 4:**
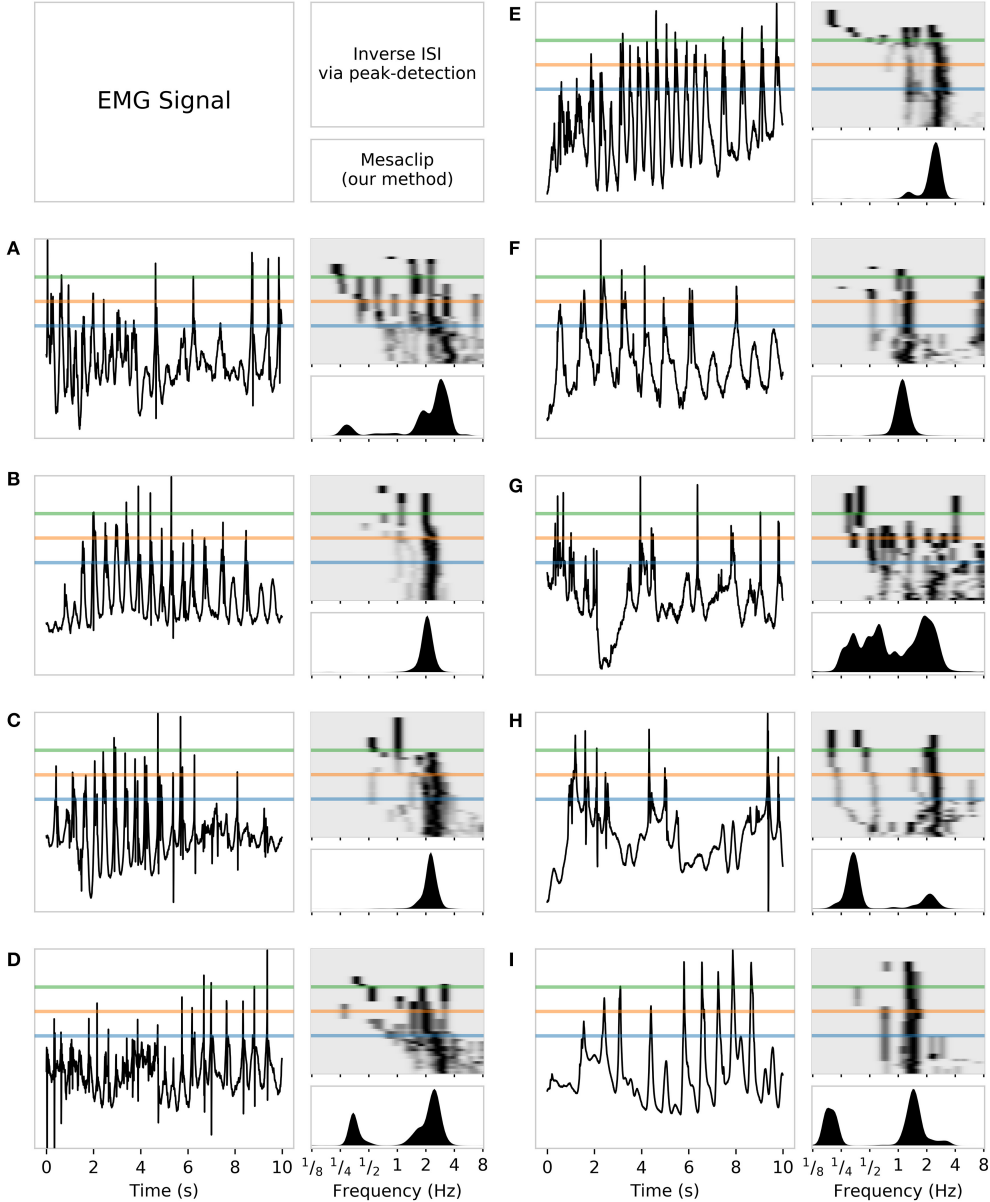
A comparison of the mesaclip method and peak-detection on EMG data from the rodent smooth-muscle, with individual recordings shown in tri-panels **(A–I)**. In each tri-panel, on the left is shown the signal of voltages from which baseline drift has been removed, and on the right are drawn results of the two approaches, where the lower corresponds to the mesaclip approach and the upper the peak approach. The result of mesaclip approach is a squared-amplitude (y-axis) per frequency (x-axis). The peak approach requires a threshold at which to identify peaks in the signal, which is represented on the y-axis going from the bottom representing the signal mean to the top representing the signal maximum. Three example thresholds of 0.3 (blue), 0.5 (orange), and 0.7 (green) are drawn highlighting those proportions of the mean-to-max level. The intensity of black represents the count of instantaneous frequencies (inverse inter-spike-intervals) scaled to the maximum per threshold. A slight Gaussian smoothing over frequency (σ = 0.06 log Hz) was performed on the results for better visualization.

We also compared the mesaclip approach to simple spike-detection based on threshold crossing, which we refer to as “peak.” The peak method works by first deciding on a threshold voltage level. Each interval of time with contiguous samples above the threshold is considered a peak. The spike time is given by the time of the maximum voltage within that peak interval. Figure 4 shows a comparison between these two approaches applied to EMG data recorded from rodent smooth muscle.

**Figure 5:**
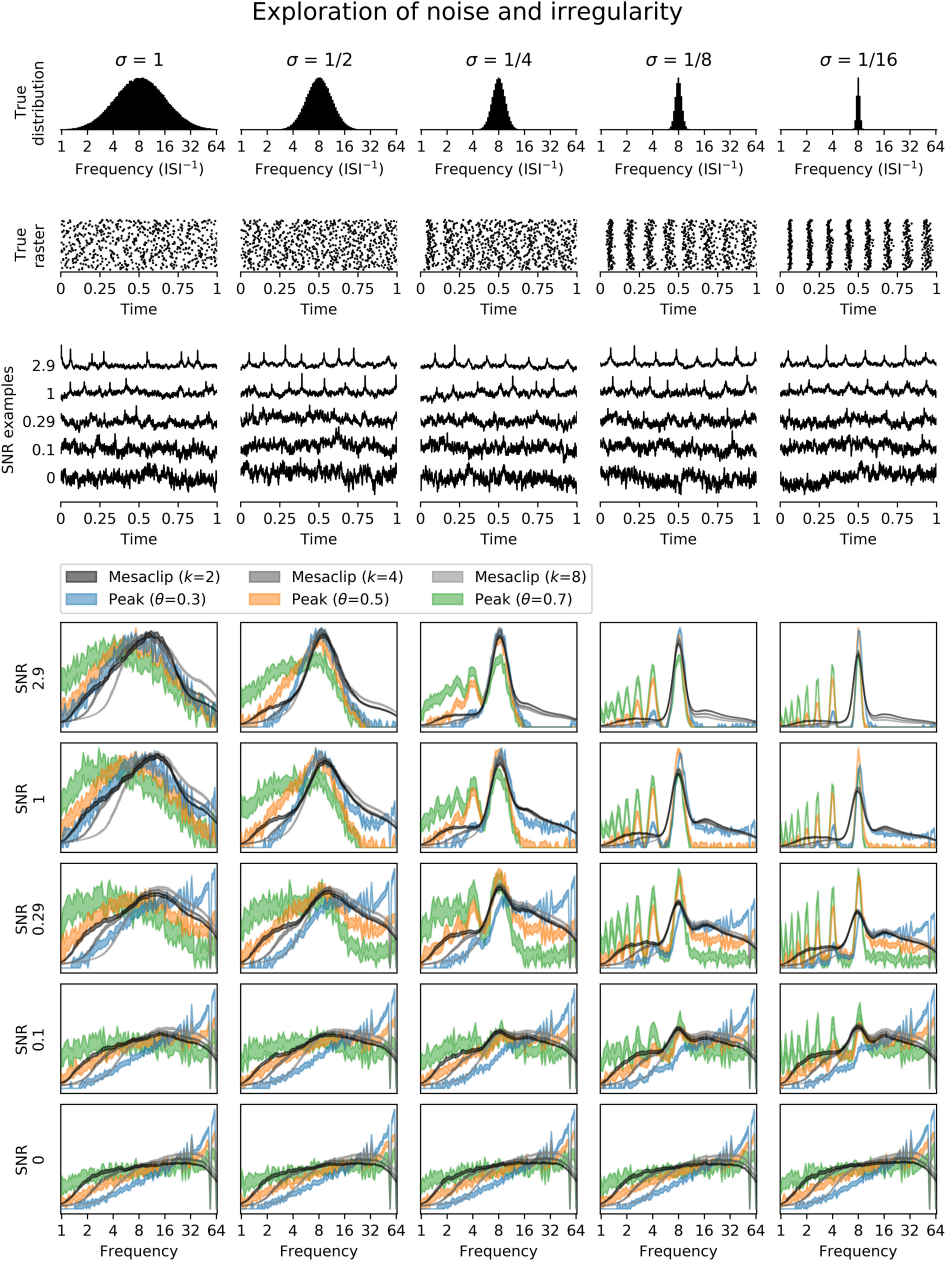
Artificial spike-trains of varying degrees of regularity (columns) and noise were generated. The top three rows show, from the top, the distribution of true instantaneous frequencies from all spike trains, a rasterization of true spikes from 100 spike-train examples, and a single spike train signal for each of the 5 signal-to-noise ratios (SNR). The lower half of the figure shows, for each SNR per row, the distribution of frequency values for each estimation method as bands of 95% bootstrap confidence intervals over the 1,000 generated spike trains. The values for the mesaclip approach are the time-averaged squared wavelet amplitudes, and for the peak approach are the histogram counts, each normalized to a sum of 1.

To explore how the mesaclip approach compares to the peak approach, we simulated signals of varying degrees of signal-to-noise ratio (SNR) and spike regularity. To generate each signal, we randomly sampled spike trains where the inverse of each inter-spike-interval (ISI) was drawn from the log-normal distribution with μ = log_2_(8) and $\sigma \in \left\{1,\frac{1}{2},\frac{1}{4},\frac{1}{8},\frac{1}{16}\right\}$. Each spike train was then rasterized into a signal with 0 where no spike occurred and 1 where a spike occurred. The signal was then Gaussian-smoothed with a width of σ = 0.01 to simulate membrane potential bumps, and a single time-sample spike was added with a noisy height drawn from 1/(1+*e*^*x*^) with x~N(0,1) to simulate spike-height variability. Pink noise (1/*f*) was added with varying degrees of SNR. For each level of regularity and SNR, 1,000 spike train signals were generated. The second and third rows of Figure 5 show examples of the spike trains and the generated signals, with the top row showing the histogram of inverse ISIs from all spike trains.

**Figure 6:**
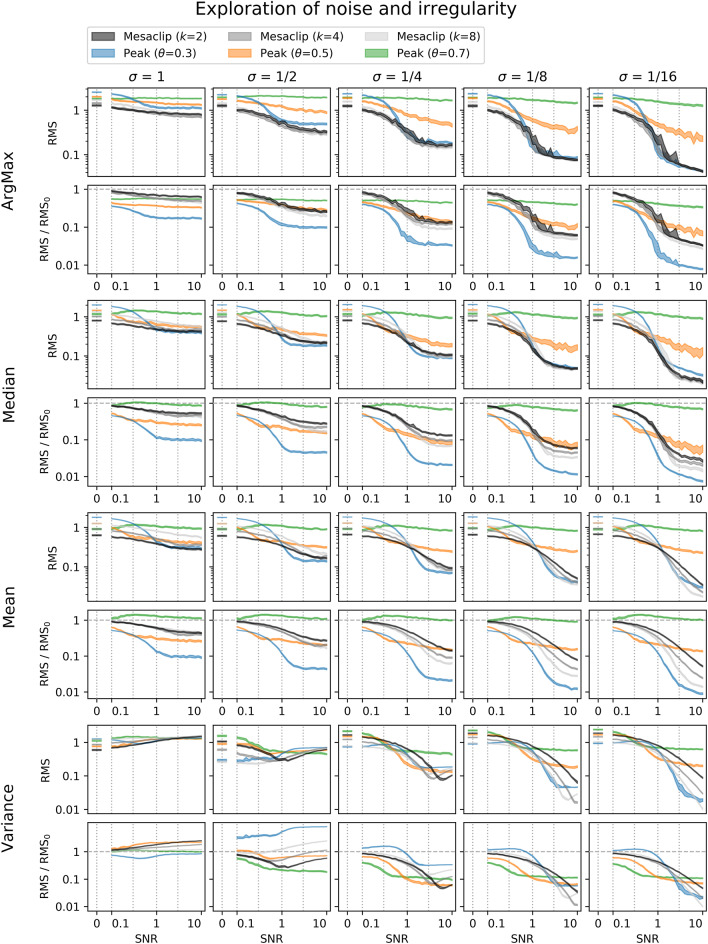
Errors in estimating various parameters of the frequency distribution of artificial spike signals, plotted over a range of signal-to-noise ratios (SNR). Columns represent different degrees of variability, corresponding to those in Figure 5. Four pairs of rows show different parameters of estimation, where ArgMax finds the frequency with maximum value, and the Median, Mean, and Variance, correspond to those estimates of frequency weighted by the values. The SNRs of (0, 0.1, 0.29, 1, 2.9) from Figure 5 are identified with vertical dotted lines. Errors are shown as root-mean-squared (RMS) in the upper of each row-pair, and since the error may be biased based on the approach, the lower row of each pair is divided by the SNR = 0 error. Bands are shown as 95% bootstrap confidence intervals of 1,000 spike train signals.

For each signal, the mesaclip algorithm was run and the global wavelet spectrum calculated by averaging the squared amplitudes over time. Although *k* = 2 is recommended, *k* = 4 and *k* = 8 have also been shown. Concurrently, the peak approach for 3-levels of threshold (with proportions 0.3, 0.5, and 0.7) between the signal's mean and its maximum were calculated, and the histogram of the inverse ISIs calculated over the same frequency domain as for the mesaclip approach. Figure 5 shows examples of the spike signals and the two frequency estimation approaches, and Figure 6 shows a comparison of errors in the frequency estimation.

## 5. Discussion

The mesaclip algorithm depends on the highly time-localized wavelet described in section 3, which results in a necessarily poorer frequency localization due to Heisenberg uncertainty (Mallat, 2008). This limitation should be taken into account with regard to the characteristics of the signal to be analyzed when deciding whether to use mesaclip, or if using mesaclip whether to apply a post-processing step to refine the frequency distribution. An analogy would be if one were to average inter-spike-intervals (ISIs) over successive time-blocks. Fewer but longer blocks would result in a tighter distribution of average ISIs, whereas a greater number of shorter blocks would result in a wider distribution of average ISIs. One could use a post-processing step to combine multiple smaller blocks to refine the averages. However, such a refinement algorithm is not covered in this paper.

The results in section 4 show how well the proposed mesaclip approach works on a variety of signals without any parameter tuning, compared to the peak approach which is highly dependent on the arbitrary choice of threshold. In particular, Figure 5 shows that the mesaclip approach works well on various signal-to-noise ratios and does not suffer from the subharmonic artifacts that the peak approach does on the simulated data. The subharmonic artifacts are due to noisy spike heights randomly falling below the chosen threshold.

Since phases are retained in the mesaclip approach, one can compute the phase-difference between two signals. Another potential approach for detecting phase-differences between two spike trains is to apply cross-correlation. However, cross-correlation is a global measure that can reveal phase-differences only in stationary signals. To apply to non-stationary signals a windowing function and choice of window size is needed. An appropriate window size depends on estimating the time scale at which the stationarity breaks down. Also, consider that phase-differences can be frequency dependent. That is, two spike trains may be offset by some phase and occur in bursts offset by a different phase that could even have opposite sign. Additionally, both subharmonic and harmonic artifacts are present in auto and cross-correlations. Accounting for all these problems and free parameters leads to a cumbersome and potentially error-prone use of cross-correlation.

The principle difference between the mesaclip and peak approaches is that the mesaclip approach is essentially an amplitude-integrated or area-under-the-curve (AUC) approach and the peak approach is an amplitude-threshold approach. The AUC of any particular spike is particularly small, and so the spikes themselves have a small contribution to the result, and we rely on the underlying membrane-potential to inform the frequencies. If the underlying membrane-potential is a poor representation of the activity one wishes to observe, for example, if there is a relatively low-amplitude but high-powered oscillating artifact together with high-amplitude but low-powered spikes, then with the mesaclip approach the oscillating artifact will drown-out the spikes. In this case, if the oscillating artifact cannot be filtered out then a peak approach should be considered instead. If the spikes are very clear (as in Figure 3D), then the peak approach is simpler and may be sufficient.

## 6. Conclusion

An algorithm for the removal of high-frequency artifacts from the amplitudes of a wavelet transform was presented. The artifacts are a result of non-sinusoidal oscillations characterized as spikes of short duration or sharp edges, separated by longer intervals. The algorithm removes these artifacts by inspecting the phase differences of wavelet amplitudes and clipping their amplitudes when the phase differences are shorter than a specified number of cycles. This results in a considerable proportion of the remaining amplitudes pertaining to the intervals between spikes, allowing clearer identification of the frequencies at which events occur.

The proposed approach: (1) contains no free parameters beyond *k* which is application-dependent, recording-independent, and due to the excellent default *k* = 2 could be considered to have no free parameters; (2) allows one to obtain phase differences at each frequency; (3) the time-window over which the signal's instantaneous information is integrated is optimal for each frequency; (4) is not susceptible to subharmonic artifacts; (5) the potential problem of harmonic artifacts have now been solved with the presented mesaclip algorithm.

## Data Availability Statement

The datasets generated for this study are available on request to the corresponding author.

## Ethics Statement

The animal study was reviewed and approved by Animal Welfare Committee of Flinders University #861-13.

## Author Contributions

LW wrote the entire software development, the manuscript, and devised the methodology. LT performed the experiments and selected the pertinent recordings. All the other authors helped to write and review, and establish the concepts presented.

## Conflict of Interest

The authors declare that the research was conducted in the absence of any commercial or financial relationships that could be construed as a potential conflict of interest.

## Footnotes

^1^Such a mapping does not exist in general due to instantaneous frequencies of 0, but we can work around this by treating ϕ^−1^(φ′) as a fiber and later replacing *r*(ϕ^−1^(φ′))≥*h* with {*t* ∈ ϕ^−1^(φ′) | *r*(*t*)≥*h*}≠∅.
